# Cefazolin Irreversibly Inhibits Proliferation and Migration of Human Mesenchymal Stromal Cells

**DOI:** 10.1155/2016/2042687

**Published:** 2016-03-16

**Authors:** Hakan Pilge, Julia Fröbel, Sabine Lensing-Höhn, Christoph Zilkens, Rüdiger Krauspe

**Affiliations:** Department of Orthopaedic Surgery, Heinrich-Heine-University, 40225 Düsseldorf, Germany

## Abstract

Drugs may have a significant effect on postoperative bone healing by reducing the function of human mesenchymal stromal cells (hMSC) or mature osteoblasts. Although cefazolin is one of the most commonly used antibiotic drugs in arthroplasty to prevent infection worldwide, there is a lack of information regarding how cefazolin affects hMSC and therefore may have an effect on early bone healing. We studied the proliferation and migration capacity of primary hMSC during cefazolin treatment at various doses for up to 3 days, as well as the reversibility of the effects during the subsequent 3 days of culture without the drug. We found a time- and dose-dependent reduction of the proliferation rate and the migratory potential. Tests of whether these effects were reversible revealed that doses ≥250 *μ*g/mL or treatments longer than 24 h irreversibly affected the cells. We are the first to show that application of cefazolin irreversibly inhibits the potential of hMSC for migration to the trauma site and local proliferation. Cefazolin should be administered only at the required dosage and time to prevent periprosthetic infection. If long-term administration is required and delayed bone healing is present, cefazolin application must be considered as a cause of delayed bone healing.

## 1. Introduction

In orthopaedic surgery, antibiotics are applied to significantly reduce the incidence of infection and osteomyelitis [1, 2]. Cefazolin is a first-generation cephalosporin, which is used as a single-shot parenteral antibiotic or is locally applied in bone cement during orthopaedic surgery [3, 4]. It is a *β*-lactam antibiotic, which inhibits cell wall synthesis of the bacteria. Cefazolin is 90% bactericidal to* S. aureus* at concentrations greater than 100 *μ*g/mL, which can be reached with a single 2 g dose [5, 6]. Notably, reports show that bone and soft tissue concentration depend on obesity/body-mass-index (BMI), patients' age, and method of application (e.g., local application with bone cement versus systemic application). Therefore, serum and local concentrations may differ and reach lower or even high and toxic levels [7–11].

Perioperative antibiotic prophylaxis in orthopaedic surgery is usually performed with a single intravenous dose at the time of anaesthesia induction [12]. If the surgery lasts more than 3-4 h or the half-life of the antibiotic is short, additional doses every 4–8 h are recommended [13] or prophylaxis longer than 24 h is performed, especially when the operating environment is poor [12].

Although cefazolin is the most commonly used antibiotic drug in arthroplasty antibiotic prophylaxis worldwide [3, 14], there exist only a few reports of how it affects human osteoblasts and its progenitor cells and, therefore, how it may affect postoperative bone healing [15, 16]. There are several reports that antibiotic drugs have a significant effect on osteoblasts. Cefuroxime, for example, showed a dose-dependent increase in proliferation and alkaline phosphatase activity of human osteoblasts* in vitro* [9]. In addition, other antibiotics, such as clindamycin and rifampicin, have* in vitro* effects on osteoblasts or their progenitor cells [10, 17].

The aim of this study was to evaluate the effect of cefazolin, the most commonly used antibiotic worldwide during arthroplasty, on hMSC. To better understand the effect of cefazolin on the early stages of bone healing, the modulation of cell migration and proliferation and its reversibility was evaluated and compared to cell cultures without drug administration.

## 2. Material and Methods

The study protocol was approved and authorised by the ethical committee of our institution. Written informed consent was obtained from all patients before surgery. During elective surgery, bone marrow was harvested from the iliac crest or the femoral head in 13 patients. Median age was 56 years (range 17 to 73) in 8 male and 5 female patients. None of the patients had a history of bone marrow pathologies.

### 2.1. MSC Isolation and Expansion

Bone marrow mononuclear cells were separated by density-gradient centrifugation (Biocoll 1.077 g/mL, Biochrom GmbH, Berlin, Germany) and seeded in growth medium (DMEM low glucose [Sigma-Aldrich, St. Louis, MO, USA], 20% foetal bovine serum [FBS Superior, Biochrom GmbH], and 1% penicillin/streptomycin/L-glutamine [Sigma-Aldrich]) in a humidified atmosphere at 37°C and 5% CO_2_. After one week nonadherent cells were removed, and growth medium was changed every 3-4 days. Adherent cells were passaged weekly and seeded at 5000 cells/cm^2^. Experiments were carried out using MSCs derived from passage 3. Before that, the MSC character of the cultured cells was determined and possible contamination with haematopoietic cells excluded by flow cytometry (≥95% expression of CD73, CD90, and CD105, while lacking CD34 and CD45).

### 2.2. MSC Migration

For each concentration and time point of drug treatment, 10^5^ trypsinized MSCs from 13 donors were resuspended in medium without drugs or FBS and were placed in cell culture inserts with 8 *μ*m pores (Greiner Bio-One GmbH). The inserts were placed in 12-well plates containing growth medium with 50 ng/mL stromal cell-derived factor 1*α* SDF-1*α* (PeproTech, Rocky Hill, NJ, USA) as a chemoattractant. After 20 h of incubation in a humidified atmosphere at 37°C and 5% CO_2_, cells that had migrated to the lower chamber were trypsinized and counted.

### 2.3. MSC Proliferation

Passage 3 MSCs were seeded at a density of 5000 cells/cm^2^ and allowed to stabilize overnight before they were cultured for 24, 48, and 72 h with 50, 100, 250, 500, or 1000 *μ*g/mL cefazolin or a negative control without antibiotics. Then cells of all 13 donors were washed with phosphate buffered saline (PBS) and trypsinized (Trypsin-EDTA solution, Sigma-Aldrich), and the cell count and viability were determined using a haemocytometer. In addition, cells treated with cefazolin for 24 or 48 h were cultured for another 72 h without antibiotics to test the reversibility of the effects.

### 2.4. Statistics

Statistical analyses were performed using GraphPad Prism (version 5.01, GraphPad Software Inc., San Diego, California). Values are reported as means with standard error of the mean (SEM). Statistical differences between groups treated with cefazolin and untreated controls were analysed using Student's *t*-test. A *p* value < 0.05 was considered significant. Asterisks are used to show the level of significance throughout the figures (^*∗*^ *p* < 0.05, ^*∗∗*^ *p* < 0.01, and ^*∗∗∗*^ *p* < 0.001).

## 3. Results

### 3.1. Cefazolin Affects MSC Migration

**Figure 1:**
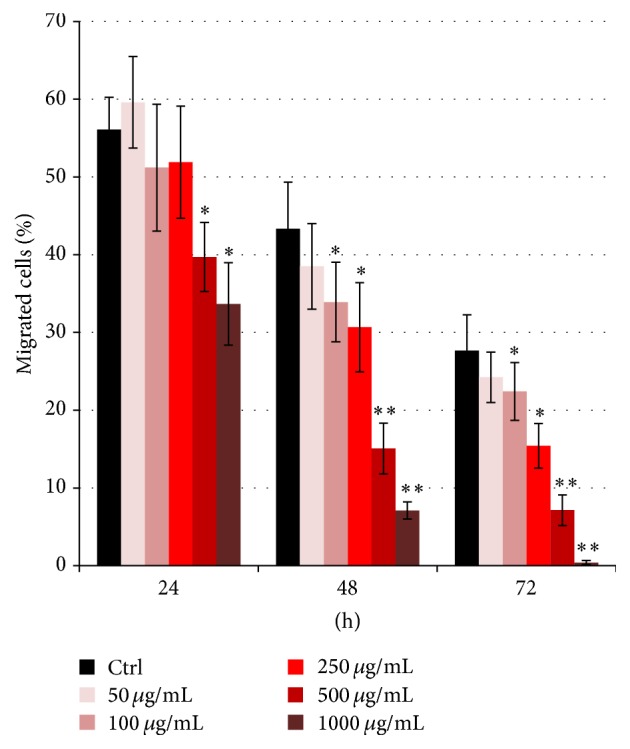
Cefazolin affects MSC migration. Bar chart showing mesenchymal stromal cells (*n* = 13) that migrated towards SDF-1. There was a significantly reduced migratory potential upon cefazolin treatment in a time- and dose-dependent manner. Asterisks show the level of significance (^*∗*^ *p* < 0.05, ^*∗∗*^ *p* < 0.01, and ^*∗∗∗*^ *p* < 0.001).

The overnight Boyden chamber migration assay was carried out immediately after each time point at which the cefazolin-treated cells and untreated controls were harvested. The longer the cells were in culture, the fewer the MSCs were able to migrate. After 24 h of culture, 56.1% of the control MSCs migrated towards the chemoattractant, while after 48 and 72 h, only 43.3% and 27.7% migrated, respectively (*p* < 0.05 for each, Table 1 and Figure 1). Furthermore, the assay revealed that cefazolin treatment of the cells resulted in a dose- and time-dependent downregulation of the migratory potential of the MSCs. After 24 h of treatment, significant effects on MSC migration were seen at 500 *μ*g/mL cefazolin and 1000 *μ*g/mL. After 48 h, migration was significantly diminished in all cells treated with 100 *μ*g/mL to 1000 *μ*g/mL cefazolin. The same effects could be seen after 72 h of cefazolin treatment.

### 3.2. Cefazolin Affects MSC Cell Count

**Figure 2:**
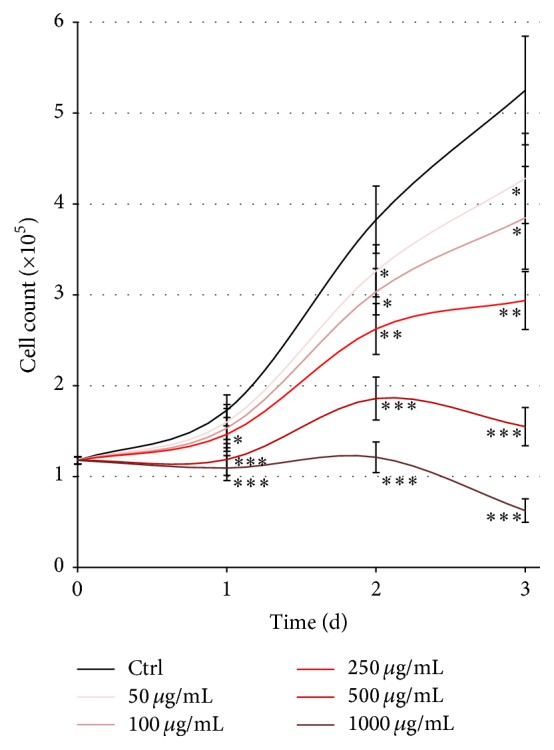
Cefazolin affects MSC proliferation. Proliferation curves showing that the cell count of mesenchymal stromal cells (*n* = 13) treated with cefazolin is significantly downregulated in a time- and dose-dependent manner. Asterisks show the level of significance (^*∗*^ *p* < 0.05, ^*∗∗*^ *p* < 0.01, and ^*∗∗∗*^ *p* < 0.001).

Studying cell lines treated with cefazolin at five different concentrations after 24, 48, and 72 h, we found a significantly downregulated dose- and time-dependent cell count (Table 2 and Figure 2). After 24 h, compared to untreated controls, MSCs treated with the three highest doses of 250, 500, and 1000 *μ*g/mL cefazolin showed a significantly lowered cell number. After 48 h and 72 h, even MSCs treated with 50 *μ*g/mL and 100 *μ*g/mL cefazolin showed a significantly reduced cell count. After 72 h of cefazolin treatment, all cells treated with cefazolin showed a significantly lowered cell count, at 1000 *μ*g/mL even lower than on day 0. This shows that long treatment at high doses not only prevents cell proliferation but also results in cell death.

### 3.3. Cefazolin Irreversibly Inhibits Proliferation and Migration of hMSC

**Figure 3:**
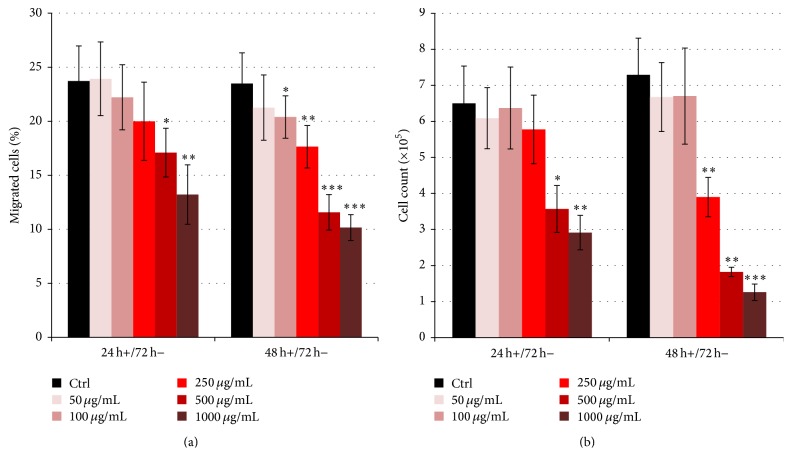
The effects of cefazolin on MSC migration and proliferation are irreversible. Bar charts showing (a) the proportion of mesenchymal stromal cells that migrated towards SDF-1 and (b) number of cells after 24 h or 48 h of cefazolin treatment with subsequent recovery for 72 h (*n* = 13). Asterisks show the level of significance (^*∗*^ *p* < 0.05, ^*∗∗*^ *p* < 0.01, and ^*∗∗∗*^ *p* < 0.001).

MSC cell lines treated with cefazolin for 24 h and 48 h were subsequently cultured for 72 h in media without the drug to give them time to recover. Proliferation and migration were determined as described above. The migration rate of cells treated for 24 h with 500 *μ*g/mL (17.1%, *p* < 0.05) and 1000 *μ*g/mL cefazolin (13.2%, *p* < 0.01) was still significantly reduced compared to the control cells (23.7%, Figure 3(a)). Cefazolin treatment for 48 h had even stronger effects that were also not compensated for during the recovery time. Compared to the migratory capacity of the control cells (23.5%), that of MSCs treated with 100 *μ*g/mL cefazolin (20.4%, *p* < 0.05), 250 *μ*g/mL (17.6%, *p* < 0.01), 500 *μ*g/mL (11.6%, *p* < 0.001), and 1000 *μ*g/mL (10.2%, *p* < 0.001) remained significantly reduced. Similarly, compared to the control MSCs (6.50 × 10^5^ cells), even after 72 h of recovery time, the number of cells treated for 24 h with 500 *μ*g/mL (3.57 × 10^5^ cells, *p* < 0.05) and 1000 *μ*g/mL cefazolin (2.91 × 10^5^ cells, *p* < 0.01) was still significantly reduced (Figure 3(b)). Treatment for 48 h with cefazolin had stronger effects on the cells. Compared to untreated cells (7.29 × 10^5^ cells), MSCs treated with 250 *μ*g/mL (3.90 × 10^5^ cells, *p* < 0.01), 500 *μ*g/mL (1.83 × 10^5^ cells, *p* < 0.01), and 1000 *μ*g/mL cefazolin (1.26 × 10^5^ cells, *p* < 0.0001) had a significantly lower cell count.

## 4. Discussion

One of the most feared complications of arthroplasty is periprosthetic infection, which develops in 0.4–2% of patients [18]. Cefazolin, a *β*-lactam antibiotic and a first-generation cephalosporin, is effective against gram-positive bacteria (e.g., staphylococci, streptococci), some gram-negative bacteria (*Escherichia coli* and* Klebsiella pneumoniae*) and is on the World Health Organization's List of Essential Medicines (19th list, April 2015). It achieves highest peak bone concentrations of all first-generation cephalosporins 40 min after parenteral application with a serum half-life of 108 min and bone half-life of 42 min [3]. It is 90% bactericidal to* S. aureus* at concentrations greater than 100 *μ*g/mL and eliminates bacteria within 48 h at concentrations of 250 *μ*g/mL or within 24 h at 500 *μ*g/mL [5]. Interestingly, there is a correlation between pharmacokinetics of antibiotics and body size measurements. Cefazolin differs in its tissue and body water distribution, which can therefore result in therapeutic failures or drug-related toxicities [7, 8]. In addition, there seems to be a significant correlation between younger age and higher cefazolin clearance [11]. Some authors recommend either systemic application of cefazolin, administration as an addition to bone cement, or local osseous application [19–21].

Bone healing is a dynamic process that is composed of stages of inflammation, repair, and remodelling. After the initial inflammatory process, MSCs are recruited to the trauma site, mediated by bone-morphogenetic-protein-7 (BMP-7), stromal cell-derived factor-1 (SDF-1), and C-X-C chemokine receptor type 4 (CXCR-4) [22, 23]. BMP-5 and BMP-6 have been suggested to induce cell proliferation [24, 25]. After migration of MSCs to the trauma site and local proliferation, the differentiation cascade into osteoblasts is initiated by the Wnt family molecules. Finally, these mature osteoblasts carry out the remodelling process, starting 3-4 weeks after surgery [22].

In this study, we focused on the migratory and proliferative potential of human MSCs as the osteoblastic progenitor cells under cefazolin treatment in different concentrations. We found that cefazolin had an inhibitory effect on cell migration, as well as proliferation, in a time- and dose-dependent manner. We showed for the first time that treatment for 48 or 72 h inhibited migration at doses of ≥100 *μ*g/mL cefazolin and decreased proliferation even at doses as low as 50 *μ*g/mL, which is not even bactericidal [5]. Doses of ≥500 *μ*g/mL cefazolin not only slowed down cell proliferation, but also led to cell death of the initial MSCs, which is in line with the effects this drug has been shown to have on osteoblasts [15]. Furthermore, we showed that the surviving cells were no longer able to migrate towards SDF-1 and, therefore, would not reach the trauma site. Our tests of whether these effects are reversible when the drug is removed showed that neither the migratory nor the proliferative potential of the MSCs recovered from cefazolin treatment. Therefore, our results show that cefazolin treatment for more than 24 h, even at low doses, negatively affects MSC migration to and proliferation at the trauma site.

Furthermore, even during the short-term treatment of 24 h, we could show that one dose of ≥250 *μ*g/mL cefazolin inhibited the migration and proliferation of the MSCs. Our reversibility tests showed that neither the migratory nor the proliferative potential of the MSCs recovered from cefazolin treatment at doses of >250 *μ*g/mL, which is the minimum recommended for* S. aureus* infections [5]. Therefore, even short-term administration of the minimum cefazolin dose of ≥250 *μ*g/mL leads to irreversible effects on MSCs, thus slowing down their migration to the trauma site, as well as their on-site proliferation. Haasters et al. showed that reduction of migration and invasion of MSC resulted in a delay in callus formation and reducing endochondral ossification in osteoporotic patients [26].

Regarding antibiotic-impregnated cement or antibacterial coating of implants, our results add to the controversial discussion of these. While in a study with 22,889 total knee arthroplasties (TKA) there was no difference of the infection rate with and without antibiotic-loaded cement [27], others showed that cefuroxime-impregnated cement in addition to systemic cefazolin for 1 week in primary TKA decreases the infection rate to 0.19% in 2700 TKA [19]. Regarding cell toxicity, still others have shown very low metabolic activity, revealing a higher cytotoxic potential of antibiotic-coated prostheses, as well as a shift from osteogenic to adipogenic cell differentiation [28]. Therefore, regarding our results, we suppose that long-term administration of cefazolin can lead to delayed bone healing due to irreversible inhibition of migration and proliferation of MSCs, especially when there are local antibiotic concentration peaks due to antibiotic-loaded cement or implants. To verify whether these effects reflect clinical outcome further clinical studies are needed.

## 5. Conclusion

In this study, we demonstrate that cefazolin has an irreversible negative effect on migration and proliferation of primary human MSCs, which are the direct progenitors of osteoblasts, and therefore may play an inhibitory role in early stages of postoperative bone healing. Our data suggest that the balance between the targeted bactericidal effects and host cellular toxicity is critical for skeletal cell survival and function. Based on this* in vitro* study, we recommend that cefazolin should be administered only at the required dosage and only for short-term use to avoid negative effects during bone healing.

## Figures and Tables

**Table 1:** Migration after preincubation with cefazolin.

Cefazolin [*μ*g/mL]	Migration rate after		
	24 h cefazolin	48 h cefazolin	72 h cefazolin
0	56.1% ± 4.2%	43.3% ± 6.0%	27.7% ± 4.6%
50	59.6% ± 5.9%	38.5% ± 5.5%	24.3% ± 3.2%
100	51.2% ± 8.2%	33.9% ± 5.1%	22.4% ± 3.7%
250	51.9% ± 7.2%	30.7% ± 5.7%	15.4% ± 2.9%
500	39.7% ± 4.4%	15.1% ± 3.3%	7.1% ± 2.0%
1000	33.7% ± 5.3%	7.1% ± 1.1%	0.4% ± 0.3%

**Table 2:** MSC proliferation under cefazolin treatment.

Cefazolin [*µ*g/mL]	Cell count after		
	24 h cefazolin	48 h cefazolin	72 h cefazolin
0	173.000 ± 17.018	382.625 ± 36.930	525.000 ± 59.806
50	160.000 ± 19.330	326.375 ± 28.588	428.125 ± 49.641
100	153.250 ± 21.659	303.438 ± 25.438	384.688 ± 56.617
250	146.500 ± 18.587	262.500 ± 28.062	293.750 ± 31.886
500	118.750 ± 17.506	185.875 ± 23.578	155.000 ± 21.044
1000	109.375 ± 13.634	121.250 ± 16.764	62.500 ± 12.956
